# Effects of first and second generation antihistamines on muscarinic induced mucus gland cell ion transport

**DOI:** 10.1186/1471-2210-5-8

**Published:** 2005-03-24

**Authors:** Huiling Liu, Jerry M Farley

**Affiliations:** 1 Department of Pharmacology and Toxicology, University of Mississippi Medical Center, 2500 N. State St., Jackson, MS 39216, USA

## Abstract

**Background:**

The first generation antihistamines, such as diphenhydramine, are fairly potent muscarinic antagonists in addition to being H1 selective antihistamines. The antimuscarinic action is often not desirable since it is in part responsible for the drying of secretions in the airways and the sedative effect. We therefore examined a number of antihistamines for antimuscarinic effects on ion transport by mucus gland cells isolated from the airways of swine. Enzymatically isolated airway mucus gland cells were purified utilizing density gradients and grown in culture on porous inserts (Millicell HA™) at an air interface. Cells grown in this manner maintain phenotype and polarity. Transport of ions, as short-circuit current measured under voltage-clamp, was measured in response to acetylcholine (ACh) or histamine applied to the serosal side of the gland cell layers. Concentration-response relationships for ACh or histamine were generated in the presence and absence of various drugs. The potencies against muscarinic receptor activation were estimated using the dose-ratio method of Schild.

**Results:**

Three known muscarinic antagonists were used to validate the system. Atropine had a pA_2 _of 9.4 ± 0.1 (n = 9). 4-DAMP and methoctramine had pA_2 _values of 8.6 ± 0.1 and 5.6 ± 0.1, respectively (n = 12, 11) all consistent with inhibition of an M3 subtype muscarinic receptor. The rank order of potency of the antihistamines against the inhibition of M3 receptors was desloratadine = diphenhydramine > hydroxyzine (pA_2_; 6.4, 6.2, 4.8, respectively). pA_2 _values for fexofenadine, loratadine and cetirizine were not determined since they had no effect on the cholinergic response at the highest drug concentrations tested (10, 10 and 100 μM, respectively). The pA_2 _values for the antihistamines against the histamine response could not be calculated, but the estimates of the rank order of potency were estimated to be desloratadine> cetirizine ≈ hydroxyzine > fexofenadine > loratadine > diphenhydramine.

**Conclusion:**

The rank order of selectivity for histamine receptors over muscarinic receptors was estimated to be cetirizine ≈ fexofenadine > loratadine > desloratadine ≥ hydroxyzine ≥ diphenhydramine.

## Background

The airways are lined by epithelium and the upper airways have mucus gland acini, all of which contribute to secretion of both water and mucus coating the surface. The epithelium forms a physical barrier to inhaled substances and, actively secretes and absorbs fluid to provide an appropriate thickness hydrated layer on the surface of the airways. The epithelium clears particulates from the airways by ciliary action and ingestion by macrophages. There is a local immune response to inhaled antigens in part through resident macrophages and dendritic cells. Epithelial function is controlled by neurotransmitters (ACh for example) and blood born substances (epinephrine, norepinephrine, hormones) and substances released from inflammatory cells (histamine and other substances from mast cells). Several serious diseases are linked to disfunction of the epithelium such as cystic fibrosis and asthma. Therefore proper functioning of the epithelium is critical for normal lung function.

Cholinergic stimulation of muscarinic receptors is known to increase mucus secretion from submucosal gland cells [1,2], fluid transport by submucosal gland cells [3], and ciliary beat frequency of ciliated epithelium [4-6]. The secretory functions are transient (ion, water and mucus), occurring for several minutes during continuous stimulation of the cells by ACh [1,7]. This synchrony makes sense from a functional standpoint since mucus that is secreted must be hydrated by secretion of fluid. The increase in cilia beat frequency caused by muscarinic receptor activation can then clear the ejected and secreted mucus.

Histamine can also stimulate the release of mucus and fluid by submucosal gland cells. The effects of ACh and histamine on short circuit current (a measurement of ion transport and therefore fluid movement) are transient, reflecting the transient nature of the increase in secretion of fluid. Stimulation of these cells by histamine probably does not support normal secretions, but represents mast cell degranulation, typically associated with the symptomatology of a pathological state such as asthma or allergies. The symptoms of airway irritation and hypersecretion are commonly treated with antihistamines except for the case of asthma, where excessive drying of the mucus membranes by first-generation antihistamines is considered a contraindication.

The antimuscarinic actions of first generation H1 selective antihistamines are well known [8]. In fact, some of the therapeutic efficacy of these drugs (e.g., drying of mucus membranes) and side effects (e.g., drowsiness, thickening of mucus, accelerated heart rate) may also be attributable to these actions. Generally, the antimuscarinic actions of the H1 selective antihistamines are undesirable, in particular in people with high blood pressure, arrhythmias or asthma. H1 selective antihistamines devoid of antimuscarinic properties should be useful in the treatment of asthma since mast cell degranulation occurs during the early phase of an asthma attack releasing histamine causing mucus secretion, inflammatory reactions in the airway epithelium, vasodilation in the mucosa and contraction of airway smooth muscle [9]. Each of these events leads to a narrowing or stiffening of the airways and increased resistance to air flow. Theoretically, H1 selective antihistamines devoid of antimuscarinic properties would decrease the pathological effects of histamine without altering the normal control of the cells in the airway by ACh at muscarinic, M3, receptors. Yanni et al. [10] suggested that an antihistamine, effective at both H1 and H2 receptors, lacking antimuscarinic actions, would be useful in the treatment of asthma. Lee et al. [11] proposed that selective H1 antihistamines could be useful in the treatment of mild to moderate asthma and have additive effects with leukotriene antagonists. Therefore, the following experiments were performed to determine the antimuscarinic actions of several antihistamines on muscarinic receptor induced increases in ion transport by mucus gland cell epithelium grown in culture or porous bottom inserts as measured using Ussing chamber methodology.

## Results

### Antimuscarinic effects

**Figure 1:**
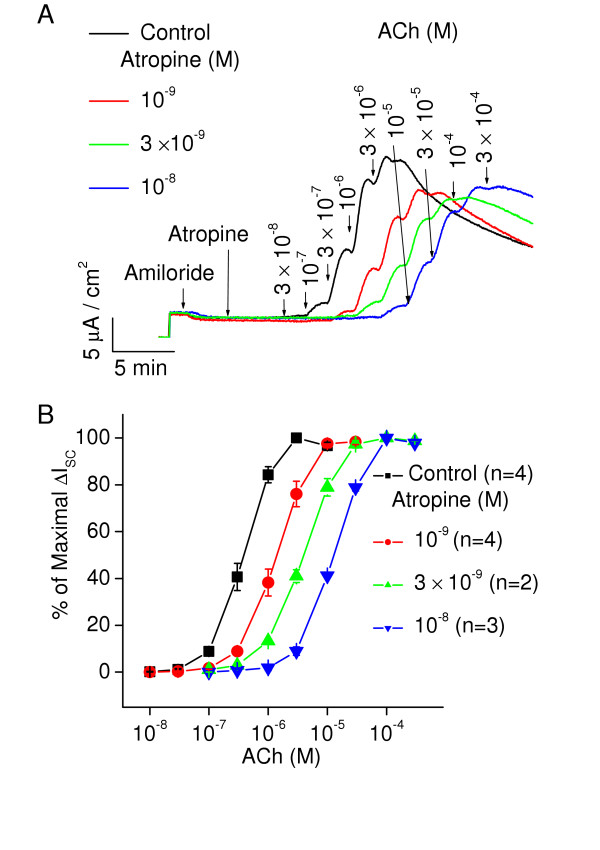
**A**. This is a recording from a typical experiment to measure short-circuit current (ΔI_sc_) across the confluent epithelial monolayers grown in Millicell inserts. Short circuit current (ΔI_sc_) is measured under voltage-clamp that maintains the potential difference across the epithelium at 0 mV. 10 μM amiloride was added in all experiments to the lumen side of the inserts to block absorption, which caused a decrease in the basal current as shown in Fig. 1A. In this recording, five minutes prior to cumulative addition of ACh, 3 inserts were exposed serosally to 10^-9^, 3 × 10^-9^, or 10^-8 ^M atropine (red, green, and blue traces respectively). The response to ACh reaches a peak and then declines. The difference of peak response to baseline line at each concentration of agonist was measured as the response to each concentration of ACh (n = 9) **B**. Concentration-response relationships were generated as the peak response to each concentration of ACh (normalized to the maximal response of the epithelium to ACh) plotted versus the ACh concentration. Atropine causes parallel shifts to the right in the concentration-response relationships with increases in atropine concentration. Average data from 9 experiments. Data were then used to generate Schild plots shown in the next figure.

**Figure 2:**
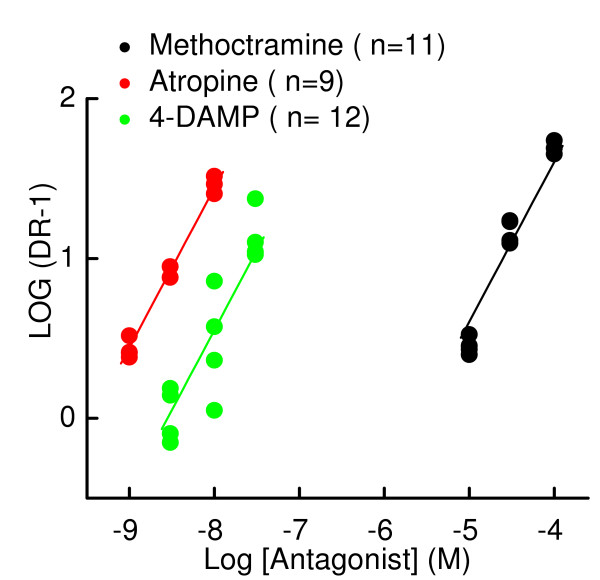
Schild analysis of the concentration response data for the muscarinic antagonists atropine, 4-DAMP, and methoctramine. The Log (DR-1) versus log inhibitor concentration is plotted in this figure. DR is the dose ratio, the ratio of concentrations of ACh giving equal responses (EC50 for this plot) in the absence and presence of test compound. The slope of the lines drawn through the data shown is 1. The X-intercepts of the lines equal pA_2_values, which are equal to the -log K_i _values, where K_i _is the affinity of the antagonist. Schild plots are shown for atropine (red) a nonselective muscarinic antagonist (n = 9), 4-DAMP (green, n = 12), M3 selective, and methoctramine (black), m2 selective (n = 11).

After two days in culture the mucus gland cells have formed confluent cell layers on the Millicell inserts and current flow can be measured across the inserts. In Figure 1A the short circuit currents (ΔI_sc_) evoked by cumulative addition of ACh are shown. The currents were used to generate concentration-response relationships for agonists on changes in ΔI_sc_(Fig 1B). The responses to both ACh and histamine are phasic, particularly obvious at higher concentrations of agonist (>10^-5 ^M). The maximum increase in current was 19 ± 1 μA/cm^2 ^(n = 21) for histamine and 15.5 ± 1 μA/cm^2 ^(n = 32) for ACh. Also, illustrated in this figure is the basic protocol used for all experiments. 10 μM amiloride is added to the mucosal side of the mucus gland cells to block fluid reabsorption. A small decrease in basal current occurs indicative of the reabsorption of sodium and fluid by the monolayer. The addition of the test drug to the serosal chamber, in this case the prototypical muscarinic antagonist atropine, causes little change in baseline current. After five minutes ACh is added cumulatively to the serosal side of the cells. The concentrations are indicated at the arrows. ACh was added at 5 to 6 second intervals into each of the 4–6 Ussing chambers being used during an experiment. Thus, the apparent "shift to the right" that occurs in the response in the presence of atropine in this figure is indicative of inhibition of the muscarinic receptors. The parallel shifts in the concentration-response relationships to the right for ACh caused by atropine are illustrated in Fig 1B for averaged peak data at each agonist concentration normalized to the maximal response for each insert. The method of Schild was used to determine the apparent K_i _for atropine as shown in Fig 2. The slope of the line through the data points is set to 1 (and not significantly different from 1) with the X intercept giving an estimate of the pA_2 _(-log K_i_). The pA_2 _for atropine (determined by this method) was 9.4 ± 0.1(n = 9). Subtype selective inhibitors of muscarinic receptors were used to suggest which subtype of muscarinic receptor was being activated by ACh to induce the increases in ΔI_sc_. 4-Diphenylacetoxy-N-methylpiperidine (4-DAMP) is a relatively selective antagonist for M3 muscarinic receptors and methoctramine is a relatively selective antagonist for m2 muscarinic receptors. The pA_2 _for these drugs was: 4-DAMP 8.6 ± 0.1(n = 12); Methoctramine 5.6 ± 0.1(n = 11). These values are consistent with M3 receptor inhibition [12]. Therefore, the inhibitory constants given for antihistamine block of muscarinic receptors are considered to be against the M3 subtype in the following experiments.

**Figure 3:**
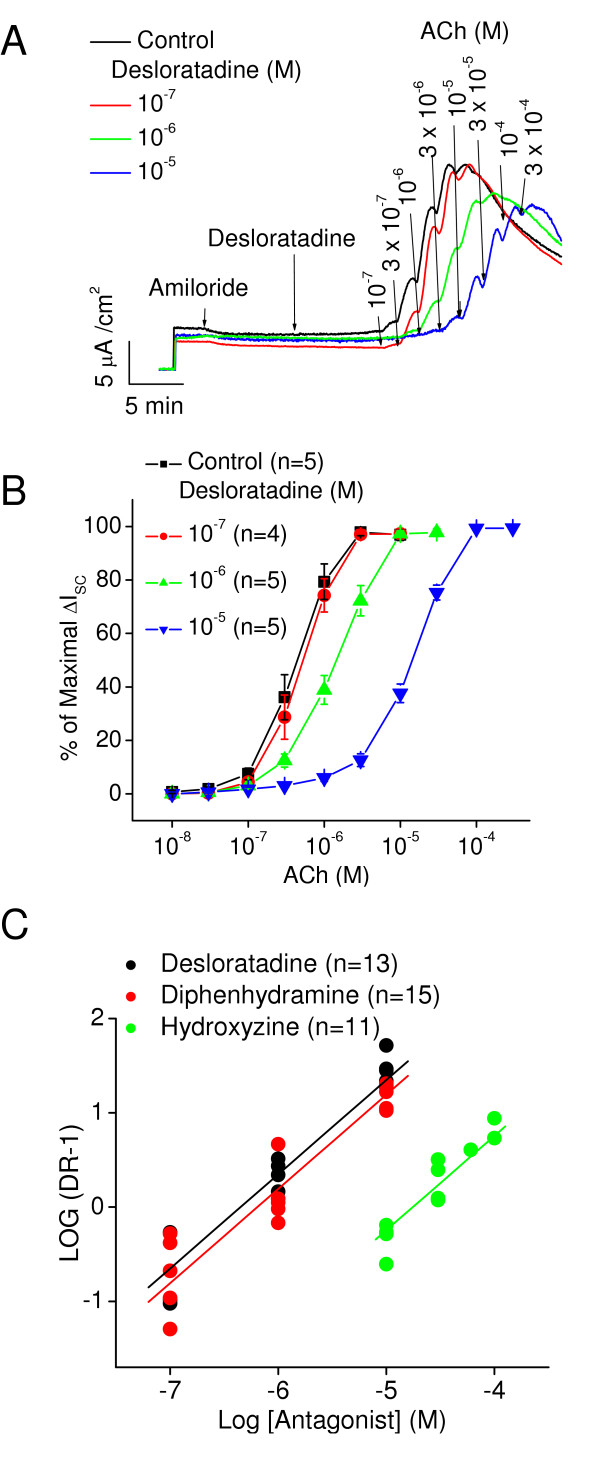
**A**. The effects of desloratadine on gland cell responses to ACh are shown here. ΔI_sc _in response to ACh are inhibited by desloratadine as shown by a shift to higher concentrations of ACh in the presence of increasing concentrations of desloratadine. **B**. The concentration response relationships derived from data in A. desloratadine causes a parallel shift to the right in the concentration response relationships for ACh. **C**. Schild analysis of these data and those for diphenhydramine and hydroxyzine are presented.

Six antihistamines were tested for inhibition of the muscarinic receptor response in gland cells. The antihistamines are: desloratadine, diphenhydramine, hydroxyzine, loratadine, cetirizine, and fexofenadine. Each was examined using the protocol described for atropine. In addition, the antihistaminic activity of each compound was confirmed by the ability of these drugs to block histamine-induced increases in ΔI_sc_. In figure 3A the effects of desloratadine on the ΔI_sc _induced by ACh are shown. As with atropine there is a shift to the right in the concentration-response relationships (Fig 3B) that, when plotted using the dose-ratio method of Schild, yields a linear plot with slope of one (Fig 3C) and an estimated pA_2 _of 6.4 ± 0.1(n = 13).

**Figure 4:**
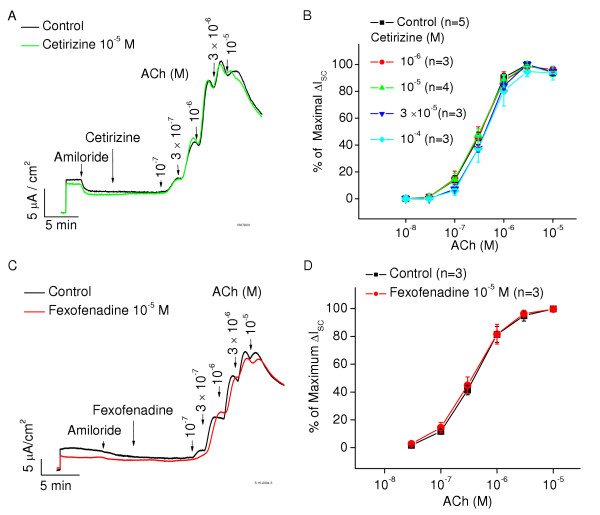
**A **and **C **show that both cetirizine and fexofenadine had no effect on the response of gland cells to ACh. **B **and **D**: Concentration-response data generated from the peak increase in ΔI_sc _induced by ACh. Note the lack of effect of cetirizine and fexofenadine.

The Schild plot for diphenhydramine and hydroxyzine is also shown in Fig 3C. Their pA_2 _values are 6.2 ± 0.1(15) and 4.8 ± 0.1(11), respectively. Hydroxyzine is much less potent antagonist at muscarinic receptors than desloratadine and diphenhydramine (p < 0.01). The active metabolite of hydroxyzine, cetirizine, has no effects on muscarinic receptor activation (Fig 4A, 4B) even at very high concentrations (10^-4^M). The precursor of desloratadine, loratadine, had no antimuscarinic action (data not shown). Fexofenadine had no antimuscarinic action at concentrations up to 10 μM (Fig 4C, 4D). A compilation of the pA_2 _values is presented in Table 1.

**Table 1:** 

Drug	^a^Muscarinic inhibition (pA_2_)	N	H1 receptor inhibition (~1/2 max-μM)	N
Atropine	9.4 ± 0.1	9	ND	
Methoctramine	5.6 ± 0.1	11	ND	
4-DAMP	8.6 ± 0.1	12	ND	
Diphenhydramine	6.2 ± 0.1	15	0.3	12
Desloratadine	6.4 ± 0.1	13	0.02	15
Hydroxyzine	4.8 ± 0.1	11	0.14	12
Cetirizine	No effect (at 100 μM)	13	0.12	14
Loratadine	No effect (at 30 μM)	3	12	8
Fexofenadine	No effect (at 10 μM)	3	0.25	11

a- all estimates for K_i _are statistically different (p < 0.001) except diphenhydramine and desloratadine (p = 0.135)

ND: Not done

### Antihistaminic effects

**Figure 5:**
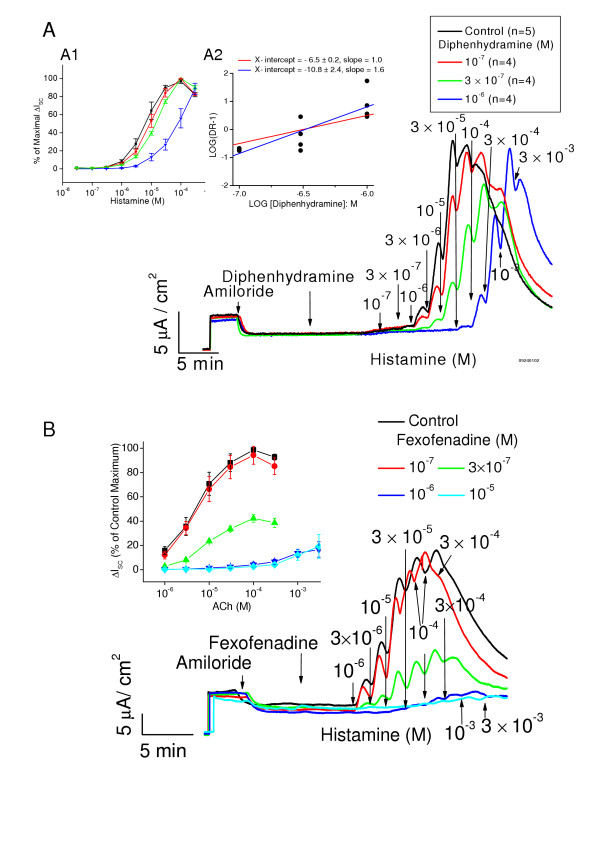
**A**. The effects of diphenhydramine on the response of gland cell epithelium to histamine. The responses to histamine, like those to ACh, are transient. However, the response to histamine decays more rapidly than that for ACh. Note that diphenhydramine causes increasing inhibition with increasing concentration of inhibitor (**A and inset A.1**) without a decrease in maximum response of the histamine action. The range of concentrations at which inhibition occurs is similar to those that inhibited the ACh response (n = 9). **Inset A.2 **shows the Schild plot generated from diphenhydramine data. Note that when the slope of the line (red) shown in the inset was set to1, the calculated pA_2 _is 6.5 ± 0.2. However the least squares fit to these data had a slope of 1.6 (blue line) with estimated X intercept of -10.8 ± 2.4, suggesting that the underlying assumptions of the Schild analysis were not met. **B**. The effects of fexofenadine on gland cell responses to histamine. ΔI_sc _in response to histamine are blocked by fexofenadine. Fexofenadine decreased the maximum ΔI_sc _and caused a small shift to the right. **Inset B **shows the concentration response relationships derived from these data (n = 8).

**Figure 6:**
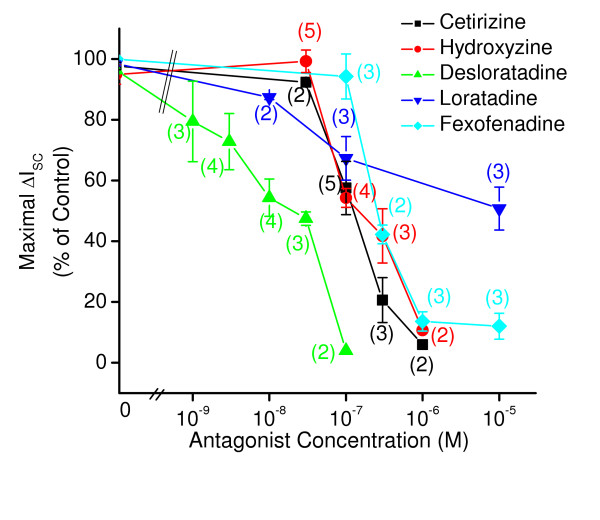
This figure shows the inhibition of histamine induced short circuit current (ΔI_sc_) by different antihistamines at various concentrations. These antihistamines concentration- dependently inhibited maximum responses induced by histamine, the order of apparent efficiencies of histamine blockade were desloratadine > cetirizine ≈ hydroxyzine > fexofenadine > loratadine.

We did not perform Schild analysis on the antihistaminic effects of the antihistamines because the underlying assumptions were not met. The basic underlying assumptions of Schild analysis are: 1) the inhibition of a response by the antagonist is competitive and 2) that during the response, equilibrium of the antagonist and agonist for the receptor is reached. While these requirements seem to be met at least tacitly for muscarinic receptors, they do not appear to be met for antagonism of the effects of histamine. Fig 5A shows the ΔI_sc _response to histamine. The ΔI_sc _response declines rapidly after reaching a peak, even at low concentrations of histamine. This provides little time for equilibrium conditions to be met during the response. In the case of diphenhydramine there is a shift to the right in the concentration-response relationships for histamine with little decrease in maximum response, as shown in Fig 5A and inset. The inset shows the resulting Schild plot with an estimated pA_2 _of 6.5 ± 0.2 (n = 12) when the slope the line shown in the inset was set to1. However, the least squares fit to these data had a slope of 1.6 (statistically different from 1 (p < 0.05) and was curvilinear with estimated pA2 of 10.8 ± 2.4. This finding suggested that the underlying assumptions of the Schild analysis were not met. This is more clearly illustrated for the case of fexofenadine as shown in Fig 5B. Fexofenadine causes a decrease in the maximum response to histamine and a shift to the right in the concentration response relationship. Using data such as shown in Fig 5B inset for inhibition of peak response, we computed the "% of maximal control ΔI_sc_" response (control inserts were run simultaneously with each drug treated insert) for each of the antihistamines; hydroxyzine, cetirizine, fexofenadine, loratadine and desloratadine. A graph of these data is shown in Fig 6. Desloratadine is the most potent antihistamine in this group (50% reduction in peak ΔI_sc _at approximately 2 × 10^-8 ^M) and loratadine the least potent (50% reduction in peak ΔI_sc _at 10^-5^M). Table 1 gives a compilation of the estimates of potency for all compounds tested.

## Discussion

In order to test the validity of using short-circuit current responses for the measurement of pA2 values for inhibition of muscarinic receptors, we first examined the effect of classical muscarinic receptor inhibitors on the ACh-induced currents. As shown in Table 1 the pA_2 _estimated for atropine was 9.4. This value is in good agreement with estimates from many other tissues (range 8.9–9.8, [12]. The pA_2 _values suggest that the muscarinic receptor primarily responsible for the increase in short-circuit current is the M3 subtype since the values estimated for methoctramine and 4-DAMP were 5.6 and 8.6, respectively. The rank order of these values is similar to those given by Caufield and Birdsall [12] for inhibition of M1 and M3 receptors, although closer to the ranges given for M3 than M1. We have previously demonstrated two receptor subtypes in submucosal gland cells, which at the time were designated M1 and M2_G _[13], corresponding to the current designations of M1 and M3. Culp et al. [14] concluded that both M1 and M3 receptors were capable of inducing secretory responses in mucus glands cells from sublingual glands and that M3 receptors were sufficient to give a maximal response. We suggest that the increase in short-circuit current is predominantly driven by M3 receptor activation, however these data do not rule out a role for the M1 subtype. These data do demonstrate the validity of this preparation as a model system for determination of the inhibition of muscarinic activity by drugs.

Fexofenadine, loratadine and cetirizine had no effect on the concentration response relationships for ACh demonstrating a lack of interaction with M3 receptors. Handley et al., [15] also found that loratadine did not bind to muscarinic receptors. All other compounds tested in our experiments competitively inhibited the increase in short-circuit current caused by ACh. Our estimate of the K_i _for hydroxyzine is 15 μM. Kubo et al. [16] found that hydroxyzine had a K_i _of 3.8 μM against muscarinic receptors in the cerebral cortex using radioligand binding assays. The estimated K_i _for desloratadine and diphenhydramine inhibition of muscarinic receptors were not statistically different (p=.135) with estimated K_i _of in the range of 0.3 to 0.6 μM. Kubo et al. [16] measured a K_i _for diphenhydramine against QNB binding in the cerebral cortex, indicative of all muscarinic receptor subtype binding, of 0.28 μM similar to our value of 0.6 μM for M3 muscarinic receptor induced secretion from mucus gland cells. Cardelus et al. [17] estimated a similar potency for desloratadine of approximately 0.2 μM (pA_2_= 6.7 ± 0.1) against muscarinic-induced contraction of rabbit iris.

By contrast, estimates of affinity using the dose-ratio method for the inhibition of the histamine receptor were not done except for diphenhydramine. The underlying assumptions of Schild analysis were clearly not met. Two of these assumptions are: 1) the inhibition of a response by the antagonist is competitive and 2) that during the response, equilibrium of the antagonist and agonist with the receptor is reached. These requirements are at least tacitly met for antihistamine binding to muscarinic receptors and one or both are clearly not met in the case of histamine receptors. The antagonists are known to bind competitively with histamine receptors as shown in receptor binding assays [16,18]. Therefore, it seems reasonable that the second condition is not met. As shown for the ΔI_sc _response to histamine in Fig 5B, the responses decline rapidly after reaching a peak (within ~15 minutes at the highest concentration the response is near baseline). The decline occurs even at low concentrations of histamine. At least quasi-equilibrium conditions need to be established within a very few minutes. Anthes et al. [18] demonstrated that for desloratadine the off-rate for binding was quite slow with only 37% of the desloratadine released from the receptor in 6 hours. Christophe et al. [19] determined the t1/2 for dissociation of cetirizine from the H1 receptor to be 142 min. The slow rate of dissociation of the antihistamine from the receptor resulted in a decreased maximum response, with little apparent shift in the concentration response relationship. This is reminiscent of non-competitive inhibition, since equilibrium cannot be reached during the transient response. Thus, the calculation of pA_2_, using the method of Schild, is not valid for antihistamine inhibition of histamine-induced increases in short-circuit current. This conclusion was also drawn by Miller et al. [20] for inhibition of the initiation of calcium transients. However, qualitatively the potencies of the antagonists can be estimated from the concentration required to reduce the peak response by 50%, as shown in Fig 5B. The rank order of potency using this method is: desloratadine > cetirizine ≈ hydroxyzine > fexofenadine > diphenhydramine > loratadine. This differs from the rank order of affinities determined by Anthes et al. [18] using radioligand binding assays: desloratadine > diphenhydramine > hydroxyzine > cetirizine > loratadine (K_b _(nM):0.9, 2.5, 10, 47, 138, respectively). Our estimates of potency are 10 to 100 times higher than the binding constants reflecting the differences in methodology. The difference in the rank order of potency primarily is due to our estimate of the potency of diphenhydramine using the dose-ratio method. We estimated a pA_2 _for diphenhydramine of 6.5 (300 nM) much higher than the K_i _estimates of 2.5–14 nM by others [16,18]. This suggests that even in the case of diphenhydramine equilibrium conditions are not met. It should be noted that the estimate of pA_2 _derived in this report assumed a slope of 1 for the Schild plot. The slope by least square fit to the data was 1.6 (p > 0.05 compared to unity) and curvilinear. Therefore, diphenhydramine inhibition is also non-equilibrium, and we expect that the actual pA_2 _for diphenhydramine is in the range of 10.8 ± 2.4 (35 pM ~3.9 nM). This conclusion was also drawn by Miller et al. [20]. They found that diphenhydramine inhibited the histamine-induced transient calcium response in an apparent non-competitive manner.

Our findings suggest that of the antihistamines tested, the rank order of selectivity for histamine over muscarinic receptors is: cetirizine ≈ fexofenadine > loratadine > desloratadine > hydroxyzine ≥ diphenhydramine. This was derived from the ratio of the estimated potencies of muscarinic inhibition and histaminergic inhibition. Since fexofenadine, cetirizine and loratadine did not affect the muscarinic response they were assumed to be the most selective with cetirizine having the higher potency toward histamine receptors.

## Conclusion

It is likely that antihistamines with significant antimuscarinic effects in this assay might show some antimuscarinic actions in vivo. However, the antimuscarinic actions probably will occur early after dosing and be short lived, since as the plasma levels of the drugs decrease muscarinic inhibition would readily reverse, compared with the comparatively slow release of antihistamines from the histamine receptors. This would be true for most second generation antihistamines and hydroxyzine, but would not be true for diphenhydramine. This is born out by the well known antimuscarinic action of diphenhydramine. Thus, of the agents tested, those with the least antimuscarinic action such as fexofenadine and cetirizine, may be the most useful for treatment of allergic rhinitis and possibly as an adjunct drug in the treatment of asthma.

## Methods

### Isolation and culture of gland cells

Tracheal submucosal gland cells were isolated from the tracheal epithelium of Yorkshire white male swine (30–40 kg) were. Animals were euthanized by exsanguination after anesthesia with 5% isoflurane. The epithelium was removed from the airway and gland cells were enzymatically dissociated and isolated on discontinuous Percoll^® ^gradients by the methods of Yang et al. [13] and Chan and Farley [21] with little modification. The cells were plated on Millicell^® ^inserts (0.04 μm pore size, 0.6 cm^2 ^area) coated with human placental collagen (20 μg/cm^2^) at a density of ~10^6 ^cells per insert in PC-1 medium. After one day in culture the medium from within the insert was removed and the cells were grown at an air interface [22,23]. As we have shown before after two days in culture the epithelium had become confluent. Experiments were performed after 3–5 days in culture.

### Measurement of short-circuit current

Inserts were mounted in Ussing chambers (Costar) modified to accept the Millicell inserts. The chambers were maintained at 37°C and continuously bubbled with 95% O_2_/5% CO_2_. The bubbling also served to drive bubble-lift circulation that quickly mixed drugs after addition to the serosal or mucosal chambers, each having a volume of 8 ml. The transepithelial short circuit current was measured using either VCC600 or VCC MC2 voltage clamp amplifiers (Physiologic Instruments) connected to the chambers via salt bridges and silver/silver chloride pellet electrodes. 10 μM amiloride was added to the mucosal solution in all experiments to inhibit sodium absorption. All other compounds (ACh, histamine, antagonists) were added to the serosal solution cumulatively from concentrated stock solutions (at least 1000× concentrated). The increases in short circuit current in response to ACh or histamine were measured as the peak currents obtained after addition of agonist at each concentration, subtracted from the baseline current measured prior to the addition of the lowest concentration of agonist. The currents were normalized to the area of the insert. Concentration response curves were generated for each insert and an EC50 determined for each insert by fitting the data with a logistic equation using Origin (Originlab). These data were then used to calculate "dose ratio – 1" values for use in a Schild plot [24] as discussed below.

### Solutions and drugs

Hepes-buffered physiological saline was used for transport, during dissection of trachea, and dissociation of cells. It contained (in mM): 140 NaCl, 5 KCl, 2 CaCl_2_, 10 Hepes; pH 7.4. A Krebs-Henseleit solution was used in all experiments in the Ussing chamber having the following composition (in mM): NaCl, 113; KCl, 4.8; CaCl_2_, 2.5; NaHCO_3_, 18; KH_2_PO_4_, 1.2; MgSO_4_, 1.2; glucose, 5.5; and mannitol, 30, pH adjusted to 7.4. This solution was bubbled with 95/5% O_2_/CO_2 _to give a pH of 7.4. Test compounds and agonists were dissolved in Krebs-Henseleit solution or DMSO. Equivalent volumes (never greater than 0.1%) of DMSO added in control experiments were without effect. All chemicals and drugs were obtained from Sigma Chemical Corporation (St. Louis, MO) except fexofenadine, desloratadine, loratadine and cetirizine, the kind gift of Aventis Pharmaceutical Corporation.

### Data analysis

Cumulative concentration-response relationships were generated by measuring the peak to baseline increases in short-circuit current induced after each concentration of ACh or histamine was added cumulatively to the serosal bath. All data are expressed as mean ± SEM. Data were normalized to the maximum peak current obtained for each insert with the particular agonist being used. Only one concentration-response relationship was generated for each insert. EC_50 _values for each agonist were determined by fitting each individual data set with a logistic function using the fitting functions in Origin™ (OriginLab). These values were then used to estimate the shift of the concentration-response relationships for ACh, by the various antagonists used. The shifts in these relationships were plotted as log (dose ratio-1) versus -log (inhibitor concentration) according to the method of Arunlakshana and Schild [24]. This should yield a plot with a slope of 1 and an X-intercept equal to pA_2_, an estimate of the antagonist affinity for the particular receptor being activated. Slopes not significantly different from unity were found for the inhibition of muscarinic receptors by all compounds. This was not true for the inhibition of histamine receptors. Quasi-equilibrium conditions must be met during the response if the dose-ratio method is to be used. These conditions are most likely never met for binding of antihistamines with the histamine receptor due to the transient nature of the response in comparison to the slow off rate for unbinding of antihistamines such as desloratadine [18]. An estimate of relative potency of the antihistamines for the histamine receptor was determined from the concentration of drug causing 50% reduction in the maximum current compared with control inserts.

## Authors' contributions

HL carried out the Ussing chamber studies and data analysis in partial fulfillment of requirements for a doctoral degree.

JMF was involved in project conception, experimental design, data analysis and manuscript preparation.
